# The *C. elegans* CEPsh glia are largely dispensable for stress-induced sleep

**DOI:** 10.17912/micropub.biology.000261

**Published:** 2020-05-30

**Authors:** Rony Soto, Cheryl Van Buskirk

**Affiliations:** 1 Department of Biology, California State University Northridge, Northridge CA USA 91330

**Figure 1:**
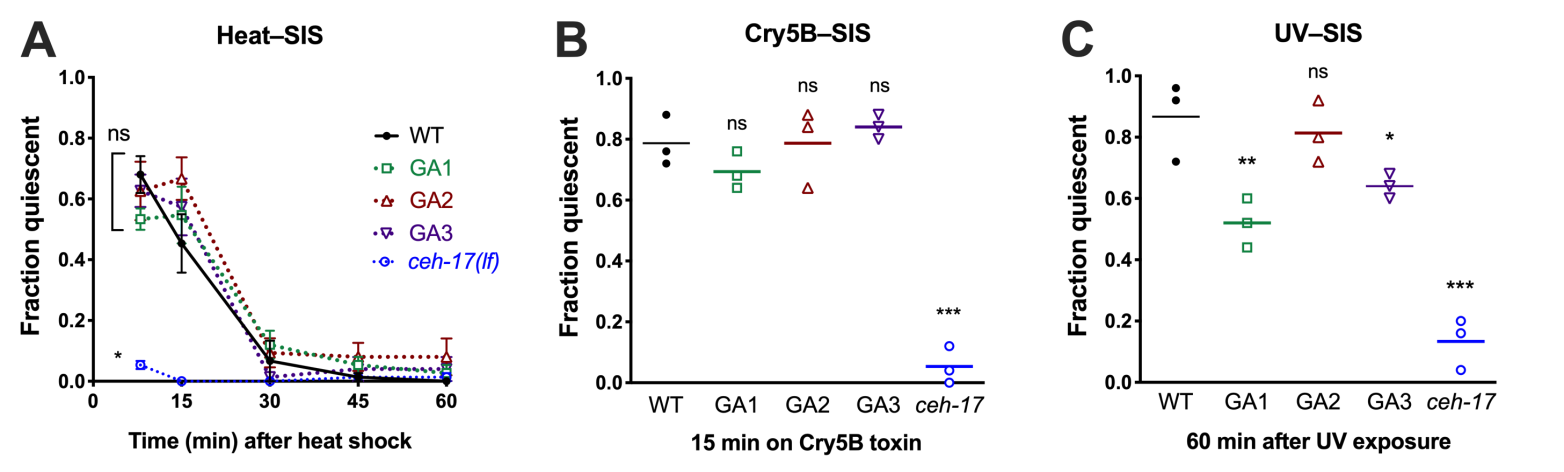
**CEPsh ablation mildly impairs UV–SIS, but heat–SIS and Cry5B–SIS are intact.** (A–C) Examination of wild-type N2, ALA–defective *ceh-17(np1),* and CEPsh glia–ablated (GA) lines during stress-induced sleep (SIS). Pre-fertile adult animals were exposed to conditions known to trigger SIS and examined for behavioral quiescence as described in Methods. Whereas reduction of ALA neuron function impairs all forms of SIS, ablation of the CEPsh glia has no significant effect on heat–SIS (A) nor Cry5B toxin–induced sleep (B). Two of the three glia–ablated lines show mildly reduced SIS following UVB light exposure (C). (A) ns = not significant, *p<0.05 vs. wild type, one-way ANOVA of area under the curve with Dunnett’s multiple comparisons test; mean and SEM of three trials of 25 animals each are shown. (B,C) ns = not significant, *p<0.05, **p<0.01,***p<0.001 vs. wild type, one-way ANOVA with Dunnett’s multiple comparisons test; each data point represents the fraction of animals quiescent in one trial (n=25), and the mean of three trials is indicated.

## Description

Across species, sleep is increased following exposure to damaging conditions, a phenomenon known as stress-induced sleep (SIS) (Hill *et al.* 2014; Lenz *et al.* 2015; Zada *et al.* 2019). In *C. elegans*, SIS is dependent on the ALA interneuron (Hill *et al.* 2014; Nelson *et al.* 2014), which promotes a coordinated quiescent state through the collective action of several neuropeptides (Nelson *et al.* 2014; Nath *et al.* 2016). Recently it has been shown that ALA ablation can suppress phenotypes associated with loss of the cephalic sensilla sheath (CEPsh) glia, such as prolonged larval development and locomotor pausing in adults (Katz *et al.* 2018), indicating that these glia attenuate certain aspects of ALA function. The CEPsh glia form a tubular structure surrounding the sensory endings of the CEP neurons and also extend thin processes that sheath the nerve ring, including the synapse between ALA and the postsynaptic AVE, a major locomotor interneuron. While the ALA is likely to promote SIS via peptidergic rather than synaptic signaling (Nelson *et al.* 2014; Nath *et al.* 2016), we wished to determine whether CEPsh glia ablation may impact the SIS-promoting function of ALA. To this end, we examined three independent lines that genetically ablate the four CEPsh glial cells (Katz *et al.* 2018), and compared the SIS responses of these strains to wild type and to ALA specification-defective *ceh-17(np1)* mutant animals. We examined three conditions known to trigger ALA-dependent sleep: noxious heat, pore-forming Cry5B toxin, and ultraviolet light exposure (Figure 1). We found that while the glia-ablated (GA) lines displayed a trend toward enhanced heat-SIS at the 15min time point, there were no significant differences in heat-SIS between the GA lines and wild-type. The GA lines also showed wild-type Cry5B-SIS. Surprisingly, two of the GA lines showed reduced UV-SIS, rather than the enhanced sleep that would be predicted if the CEPsh glia attenuate ALA’s peptidergic function. Our data indicate that CEPsh glia are largely dispensable for stress-induced sleep, but may modulate the UV–SIS response. As UV exposure elicits a delayed sleep response relative to other SIS triggers, we speculate that UV damage may activate ALA in a manner that is distinct, and partially supported by glial function.

## Methods

CEPsh glia were ablated at the L1 stage by expression of a reconstituted caspase-3 gene from the *hlh-17* promoter (Katz *et al.* 2018). Behavior was assessed in well-fed (on *E. coli* OP50) pre-fertile adult animals on NGM agar plates. Quiescence was defined as complete immobility and lack of pharyngeal pumping during a 5 second observation under a stereomicroscope at 375x magnification with experimenter blind to genotype. We observed that immobility was accompanied by feeding quiescence in all cases, but not vice versa, i.e., ‘fraction quiescent’ is equivalent to ‘fraction immobile’. For heat-SIS, 12 ml agar plates were sealed with parafilm and placed upright in a 37°C water bath for 11 minutes, then placed on ice for 2 minutes to return them to room temperature (Goetting *et al.* 2018). For Cry5B-SIS, animals were placed onto NGM plates containing Cry5B-expressing bacteria (Hill *et al.* 2014) and examined for SIS 15 minutes later in the presence of Cry5B. For UV-SIS, plates were placed lid-side down on a 302 nm 60 mW ultraviolet (UVB) light source for 50 seconds and examined 60 minutes later for SIS, as UV-induced sleep takes longer to set in than other forms of SIS (DeBardeleben *et al.* 2017; Goetting *et al.* 2018).

## Reagents

Wild-type N2 and IB16 *ceh-17(np1)* from the CGC; OS3537 (GA1) nsIs168 (P*hlh-17*::recCaspase-3, P*unc-122*::GFP, P*ptr-10*::myrRFP), OS3540 (GA2) nsIs171 (P*hlh-17*::recCaspase-3, P*unc-122*::GFP), and OS3549 (GA3) nsIs180 (P*hlh-17*::recCaspase-3, P*unc-122*::GFP) from M. Katz; Cry5B toxin from Raffi Aroian. All reagents are available from our lab.
